# Motivation in second language acquisition: A bibliometric analysis between 2000 and 2021

**DOI:** 10.3389/fpsyg.2022.1032316

**Published:** 2022-11-08

**Authors:** Xue Wu

**Affiliations:** School of Foreign Languages, Huazhong University of Science and Technology, Wuhan, China

**Keywords:** motivation, bibliometric analysis, L2MSS, SLA, multilingualism

## Abstract

As one of the most important constructs of individual differences in second language learning, motivation has garnered a lot of attention in the area of Second Language Acquisition (SLA). Nevertheless, little bibliometric research has been conducted to provide a systematic overview of this line of research, which will help researchers to better understand how motivation-related research in SLA has evolved over the past 22 years and where it might push the boundaries of SLA research into in the future. In this study, three types of bibliometric analyses (i.e., co-citation analysis, citation analysis, and keyword analysis) were performed to identify the prominent scholarly documents, authors, venues of publications, and research topics that have been highly influential in the research of motivation in SLA between 2000 and 2021. Results from scientific network maps and keyword analysis suggest significant changes in the topic over the past 22 years. The results in this study also indicate an evident continuity of theoretic development in L2 language learning motivation research. Moreover, an air of active use of qualitative approaches has been detected in L2 language learning motivation research in the last 22 years.

## Introduction

Motivation is of great importance in second language acquisition. For one thing, second language learners cannot be immersed in an ideal language learning environment where they have enough opportunities to use English and communicate with native English speakers. Therefore, motivation serves as the driving force which sustains the learning of the second language even when there is a lack of appropriate language learning environments. For another, as Gardner (2007) pointed out that compared to the learning of other subjects, which involves elements common to one’s own culture, learning a second language involves taking on elements of another culture, for instance, vocabulary, pronunciation, and language structure.

As one of the most important constructs of individual differences in second language learning, motivation has garnered a lot of attention in the area of Second Language Acquisition (SLA), and an unparalleled publication surge has been witnessed in the past few years. Several publications have offered comprehensive overviews of the constructs and their development of motivation in language learning (e.g., Dornyei and Ushioda, 2011; Boo et al., 2015). Nevertheless, little research has been conducted to provide a bibliometric overview of this line of research, which will help researchers to better understand how motivation-related research in SLA has evolved over the last years and where it might push the boundaries of SLA research into in the future.

Inspired by Zhang (2020), the present study used bibliometric approach to perform citation analysis, co-citation analysis, and keyword analysis to review publications on L2 language learning motivation. With the assistance of the mathematical and statistical methods, bibliometric analysis can extract patterns from publications which reveal the characteristics or patterns of publications in a specific discipline. In addition, bibliometric analysis can also be used to offer visualizations of the intellectual structure of the field *via* network mapping techniques. Moreover, by comparing the frequency of keywords in different periods, some significant changes to the key topics can be identified in a field (e.g., Lei and Liu, 2019; Zhang, 2020).

Based on bibliometric data retrieved from WoS from 2000 to 2021, this bibliometric study attempts to address the following four questions:

1. What are the most-cited documents published in the past 22 years?
2. What are the highly influential publications, authors and sources of publications in the intellectual structure of the L2 motivation research?
3. What are the changes of the themes and topics in motivation research?
4. What might be the directions that would be worthy of future research?

## Materials and methods

### Data

The bibliometric data used in this study were retrieved from Web of Science (WoS). According to Zhang (2020), WoS outperformed the other two major online data resources (i.e., Scopus and Google Scholar) in bibliometric analysis for three reasons. First, the amount of subscribers of WoS was two times larger than that of Scopus, making WoS a more widely used library resource. Second, both academic citations and non-academic citations were provided by Google Scholar which made it difficult to calculate the scholarly values of the publications. Third, co-citation analysis is one of the important bibliometric analyses used in this study, but co-citation information is not available in Google Scholar.

Since the present study focuses motivation research in the field of SLA, research articles published in high-quality journals in this field were included. Only published research articles were included for the reason that the quality and reliability of the unpublished preprints cannot be guaranteed due to a lack of a strict quality control mechanism such as peer review (Zhu and Lei, 2022). As for the selection of the high-quality journals, many research used the list of 15 journals provided by VanPatten and Williams (2002) to investigate issues in SLA. However, the current study adopted 16 SSCI-indexed international journals in SLA provided by Zhang (2020) for their rigorousness in peer-review processes and accessibility and visibility to the worldwide academia.

The starting year of the retrieved publications was set in 2000 because in that year Ryan and Deci (2000a,b) had conducted two momentous review studies which discussed the importance of social contextual conditions for motivation research. In one of the review articles, the authors not only retrospected classic definitions of intrinsic and extrinsic motivations, but also argued that “social contextual conditions are the basis for one maintaining intrinsic motivation and becoming more self-determined with respect to extrinsic motivation” (Ryan and Deci, 2000a, p. 65). In another article, Ryan and Deci (2000b) reviewed studies that adopted the approach of Self-Determination Theory (hereafter SDT) to investigate human motivation, and concluded that SDT could be effectively used to address the perennial debate on the activity or passivity of human beings. The two publications are quite influential (by the day this paper was written, the two review articles have received more than 20,000 citations in Web of Science) since they provided evidence of the power of social conditions on motivated behaviors and identified new directions in motivation research.

The finalized search strategy used for data retrieval in the present study was TS = (motivation) AND PY = (2000–2021) AND SO = (Applied Linguistics OR Applied Psycholinguistics OR Bilingualism Language “and” Cognition OR Computer Assisted Language Learning OR ELT Journal OR Foreign Language Annals OR Journal of Second Language Writing OR Language learning OR Language Learning “and” Technology OR Language Teaching Research OR Modern Language Journal OR ReCALL OR Second Language Research OR Studies in Second Language Acquisition OR System OR TESOL Quarterly) AND Type of publication: (Article).

### Data cleaning

Data downloaded from WoS were subject to coding errors. To guarantee the reliability of the data, the current study adopted the methods used by Zhang (2020) for data cleaning. Specifically, different author names that referred to the same author were recoded. For instance, “Dörnyei,” “Dörnyei Z.,” “Dörnyei Z,” and “Dörnyei Zoltán” were all recoded to “Dörnyei Z.” In addition, different keywords that referred to the same concept were also recoded. For instance, “mobile assisted language learning (MALL),” “mobile-assisted language learning (MALL),” and “MALL” were all recoded as “mobile assisted language learning.” Similarly, singular and plural forms of the same concept were also recoded. For instance, “possible self” and “possible selves” referred to the same concept; hence, all “possible selves” were recoded to “possible self.” It should be noted that keywords that share a degree of similarity would not be recoded since their meaning can be different. For instance, “motivational profiles” and “motivational factors” were not recoded as one since the former focused on a detailed description of a language learner’s motivation (Thompson and Vásquez, 2015), while the latter examined the various variables which may explain why a learner studies and continues to study a foreign language. For instance, issues like “how much effort they put into learning, how long they persist at learning, and how successfully they learn a language” (Ushioda, 2009, p. 218) might be discussed in research on “motivational profiles,” and topics like “why some students persist in foreign language study and others do not” (Ramage, 1990, p. 192) are more likely to appear in research on “motivational factors.”

Except for those measures, this study also checked and recoded the same abbreviations that referred to different concepts. For instance, SEM was found to be used as an abbreviation for Structural Equation Modeling, a multivariate, hypothesis-driven technique, and the Socio-Educational Model formulated by Gardner (1985) to investigated learners’ attitudes toward the TL community.

### Data analysis

Citation analysis, co-citation analysis, and keyword analysis were performed in this study. The 22-year data were divided into two periods (i.e., the 2000–2010 period and the 2011–2021 period) and the results of all the three bibliometric analyses in one period will be compared against the other period to reveal important changes during the last 22 years.

Citation analysis used citation information downloaded directly from WoS and the tool of BibExcel (Persson et al., 2009) was used to detect the impact of the documents. Specifically, the lifetime citation count of a document was retrieved from WoS, and a normalized citation is then adjusted for the time effect (Zhang, 2020).

Co-citation analysis was based on the references in the surveyed articles. The articles published in the 16 journals between 2000 and 2021 cited more than 21,000 unique references. It is hardly possible to interpret the nodes if all the cited references were included in a network map. Therefore, when constructing the network maps in VOSviewer (Van Eck and Waltman, 2010, 2017), a cutoff point was set to limit the number of nodes in a map (McCain, 1990; Zhang, 2020). For instance, a cutoff point of 13 citation counts was used to restrict the total number of the nodes to the top 50 most-cited authors when building the author network map in the 2000–2010 period. Following Zhang (2020), the cutoff points were set at the values to restrict the total number of the nodes to the top 50 most-cited items or authors in the maps for all the three types of co-citation analyses (i.e., most-cited sources of publications, most-cited authors, and most-cited publications). It should be noted that setting such cutoff points may rule out those latest research which may open up new avenues for future research.

Keyword analysis was conducted using the following procedures. First, author-supplied keywords and *n*-grams of up to four words in length and monograms of all nouns in the abstracts were extracted from the downloaded bibliometric data. It should be noted that those *n*-grams and monograms extracted from abstracts can append important research topics which may be overlooked in the author-supplied keywords (Lei and Liu, 2019; Zhang, 2020). Additionally, only monogram nouns were extracted from the abstracts for the reason that “individual adjectives, adverbs, and verbs do not constitute research topics” (Lei and Liu, 2019, p3). Second, frequency counting of each keyword was performed. In this step, if there were multiple occurrences of a keyword in an abstract, they were counted as one. Third, the log-likelihood (LL) test was conducted for the identification of significant cross-period differences of the keywords in terms of frequency in the next step. Moreover, Bayes Factor (BIC) of each keyword was also calculated using an online calculator in the following link.^1^ The LL test and the BIC were used in this study since they were widely used in corpus linguistics and bibliometric studies to identify important keywords (Rayson and Garside, 2000; Wilson, 2013; Zhang, 2020). Last, a LL value of 3.84 and a BIC value of 2 were used as criteria to determine whether there was a significant cross-period difference of the frequency of a keyword. Specifically, a word/phrase with a LL value greater than 3.84 suggested that the topic had experienced a significant change from one period to the other. Otherwise, it suggested that the interest toward this topic remained relatively stable during the two periods. BIC values supplemented the identification of keywords to LL values with a stricter criterion. Similar to Zhang (2020), BIC values greater than 2 were interpreted as an increasing/decreasing interest toward the topic with positive evidence, BIC values greater than 6.84 were interpreted as an increasing/decreasing interest toward the topics with strong evidence, and BIC values greater than 22.22 were interpreted as an increasing/decreasing interest toward the topic with very strong evidence.

## Results and discussion

The bibliometric information of a total of 752 articles was analyzed. In this section, results of the citation analysis, co-citation analysis, and keyword analysis will be presented in sequence.

### Citation analysis

The citation information of the top 10 most-cited articles in the 16 journals is presented in Table 1. The normalized citations of the articles in the second period are relatively higher than those in the first period. This result echoes findings in previous research that there is a substantial research interest in the topic of L2 motivation (Boo et al., 2015).

**Table 1:** The most-cited articles in the journals (order by normalized citation).

	2000–2010	2011–2021
	Documents	Raw Citation	Normalized Citation	Documents	Raw Citation	Normalized Citation
1	Noels, K. A., et al. (2000). Why are you learning a second language? Motivational orientations and self-determination theory. Language Learning.	361	4.52	Plonsky, L. & Oswald, F. L. (2014). How Big Is Big? Interpreting Effect Sizes in L2 Research. Language Learning.	587	12.59
2	Laufer, B. & Hulstijn, J. (2001). Incidental vocabulary acquisition in a second language: The construct of task-induced involvement. Applied Linguistics.	423	3.68	Li W. (2018). Translanguaging as a Practical Theory of Language. Applied Linguistics.	382	11.94
3	Peng, J-E. & Woodrow, L. (2010). Willingness to Communicate in English: A Model in the Chinese EFL Classroom Context. Language Learning.	188	3.66	Zou, D., et al. (2021). Digital game-based vocabulary learning: where are we and where are we going? Computer Assisted Language Learning.	42	11.86
4	Lorenzo, F., et al. (2010). The Effects of Content and Language Integrated Learning in European Education: Key Findings from the Andalusian Bilingual Sections Evaluation Project. Applied Linguistics.	185	3.60	Derakhshan, A., et al. (2021). Boredom in online classes in the Iranian EFL context: Sources and solutions. System.	39	11.02
5	Papi, M. (2010). The L2 motivational self-system, L2 anxiety, and motivated behavior: A structural equation modeling approach. System.	180	3.50	Barry, B., et al. (2021). Understanding Hong Kong primary school English teachers’ continuance intention to teach with ICT. Computer Assisted Language Learning.	30	8.47
6	Magintyre, P. D. (2007). Willingness to communicate in the second language: Understanding the decision to speak as a volitional process. Modern Language Journal.	193	3.44	Wang, N., et al. (2021). Blended learning for Chinese university EFL learners: learning environment and learner perceptions. Computer Assisted Language Learning.	25	7.06
7	Yashima, T. (2002). Willingness to communicate in a second language: The Japanese EFL context. Modern Language Journal.	419	3.40	Golonka, E. M., et al. (2014). Technologies for foreign language learning: a review of technology types and their effectiveness. Computer Assisted Language Learning.	328	7.03
8	Mills, N., et al. (2006). A reevaluation of the role of anxiety: Self-efficacy, anxiety, and their relation to reading and listening proficiency. Foreign Language Annals.	148	3.39	Boo, Z., et al. (2015). L2 motivation research 2005–2014: Understanding a publication surge and a changing landscape. System.	196	6.33
9	Dörnyei, Z. (2003). Attitudes, orientations, and motivations in language learning: Advances in theory, research, and applications. Language Learning.	255	3.31	Doernyei, Z. & Chan, L. (2013). Motivation and Vision: An Analysis of Future L2 Self-Images, Sensory Styles, and Imagery Capacity Across Two Target Languages. Language Learning.	184	6.09
10	Liu, M. & Jackson, J. (2008). An exploration of Chinese EFL learners’ unwillingness to communicate and foreign language anxiety. Modern Language Journal.	213	3.27	Rafiee, M. & Abbasian-Naghneh, S. (2021). E-learning: development of a model to assess the acceptance and readiness of technology among language learners. Computer Assisted Language Learning.	20	5.65

In the 2000–2010 period, many research topics have been examined. For instance, Liu and Jackson (2008) examined the topic of the unwillingness to communicate in Chinese English as a foreign language context, Lorenzo et al. (2010) focused on the pedagogic approach of Content and Language Integrated Learning (CLIL), and Mills et al. (2006) assessed self-efficacy beliefs and their relation to foreign language proficiency. Among the top 10 most-cited articles are empirical studies carried out to test the then emerging theoretical models. For instance, Peng and Woodrow (2010) and Yashima (2002) adopt the Willingness to Communicate (henceforth WTC) model to investigate the indirect influence of motivation on WTC in the Chinese English as a Foreign Language and the Japanese English as Foreign Language contexts, respectively. The results of these two researches not only validated the model in different cultural contexts but also identified variables which had direct or indirect influence on motivation. For example, Peng and Woodrow (2010) identified the direct effect of learner beliefs on motivation and confidence, and suggested that individual and contextual variables might be drawn on to account for classroom communication. Similarly, Papi (2010) initiatively tested a theoretical model subsuming variables in L2 Motivational Self-System (henceforth L2MSS), and found that the ideal L2 self and the L2 learning experience can ease students’ English anxiety, while ought-to L2 self-made students more anxious.

Another feature of the top 10 most-cited articles in this period is that new theories from other disciplines are introduced to the L2 motivation field. For instance, the self-determination theory (hereafter SDT) featured by empirical research in psychology was used to investigate motivation in L2 language learning by Noels et al. (2000) and Joe et al. (2017). The introduction of SDT to L2 motivation research is of great importance since it, to some degree, settled the dispute on the validity and reliability of a scale of two important types of motivation (i.e., intrinsic and extrinsic motivation) for L2 learning. The three review articles among the top most-cited articles in this period are also worth noticing. Laufer and Hulstijn (2001) investigated the cognitive and motivational aspects of L2 learning in previous literature. Macintyre (2007), based on the literatures on language anxiety and language learning motivation, argued that the decision to speak was a volitional and dynamic process. Dörnyei (2003) presented an overview of recent advances in research on motivation to L2 language learning and created the theoretical context of the selected articles.

Similar to the diversity of research topics discussed in the top-10 most-cited articles in the 2000–2010 period, different themes have also emerged in the top-10 most-cited articles in the 2011–2021 period. However, some unique features are identified in this period.

First, large datasets of publications are used in review articles. Except for Zou et al. (2021), more than 350 publications are included in the other three review articles. Specifically, Boo and collaborators reviewed 416 journal articles and book chapters published between 2005 and 2014, Plonsky and Oswald (2014) extracted and analyzed L2 effects from 346 primary studies and 91 meta-analyses, and Golonka et al. (2014) summarized evidence for the effectiveness of the use of technology in foreign language learning and teaching from over 350 studies. Second, variables related the L2 language learning environment in line with the times are investigated. For instance, prompted by the worldwide COVID-19 health crisis, online English education is reshaping L2 language learning environment. Derakhshan et al. (2021) explored causes of and solutions to boredom of online English classes in Iran, whose findings can help teachers to improve students’ experience of online English learning during and beyond the COVID-19 pandemic. In another top-10 most-cited article in this period, Wang et al. (2021) surveyed L2 language learners’ perceptions of their blended learning in a Chinese English as a foreign language context and argued that an efficient EFL learning environment can be created *via* a blended design. Moreover, new educational technologies are frequently used in L2 language learning environment (for example, the use of WeChat moments in Wang and Jiang, 2021 and the online use of English in Lamb and Arisandy, 2020). Therefore, it is not surprising to see that the topic of technology-based English as a foreign language occupies a crucial position among the top-10 most-cited articles in the 2011–2021 period. For example, Bai et al. (2021) proposed a model which involved motivational beliefs to investigate L2 language teachers’ continuance intention to use information and communication technology in their teaching, and Rafiee and Abbasian-Naghneh (2021) identified the factors which affected e-learning acceptance and readiness in the foreign language learning context.

Last, with regard to theoretical development, less empirical research has been conducted to test the theoretical hypothesis in the 2011–2021 period compared to the top-10 most-cited articles in the 2000–2010 period. The only one is conducted by Dörnyei and Chan (2013) to test the hypothesis that the students’ capability to generate mental imagery is partially determined by their intensity of motivation. The findings of Dörnyei and Chan (2013) have been widely cited by current research to examine the potential interaction between motivation or variables that are directly/indirectly related to motivation and self-images. For instance, Fathi and Mohammaddokht (2021) investigated two foreign language learning emotions (i.e., enjoyment and anxiety) as the predictors of ideal L2 self. Another example is Safranj et al. (2021) who conducted a research to determine the degree to which the ideal L2 self can be regarded as a significant factor with regard to its power to make a difference in students’ actual motivated behavior in L2 communication. Moreover, theories rooted in the field of SLA have been developed. For instance, Li (2018) developed Translanguaging as a theory of language which emphasized the multimodal and multisensory nature of the social interaction of foreign language learners as multilingual language users.

### Co-citation analysis and network mapping

Results from the citation analysis shed light on identifying the most-cited publications in the field of L2 motivation research as independent units. In this section, the highly influential references, authors, and sources of publications in the intellectual structure of the L2 motivation research are presented and discussed based on the results of co-citation analysis and network mapping.

#### The highly influential references

**Figure 1:**
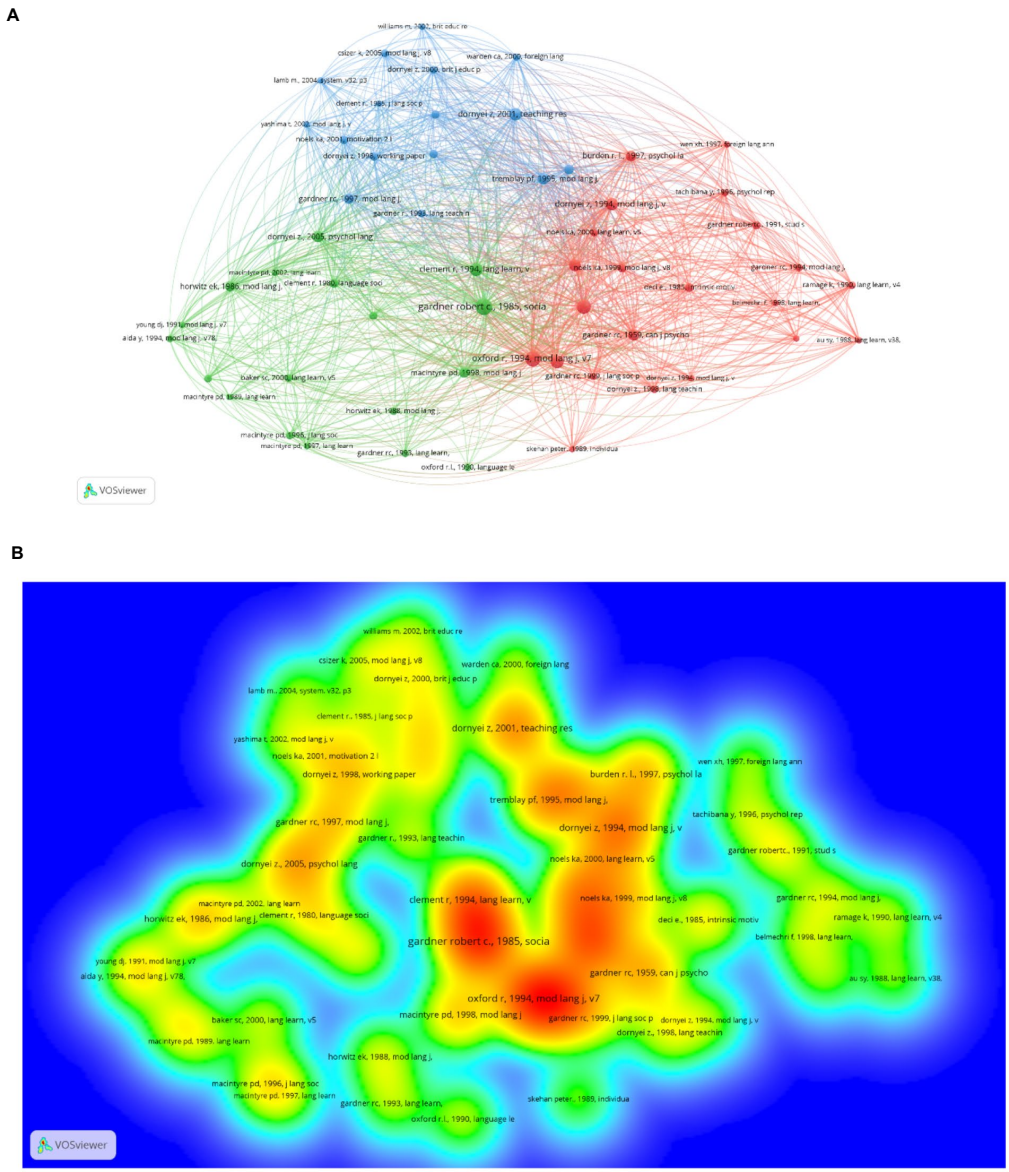
**(A)**. Network map of the most-cited references (2000–2010); **(B)**. Density view (2000–2010).

**Figure 2:**
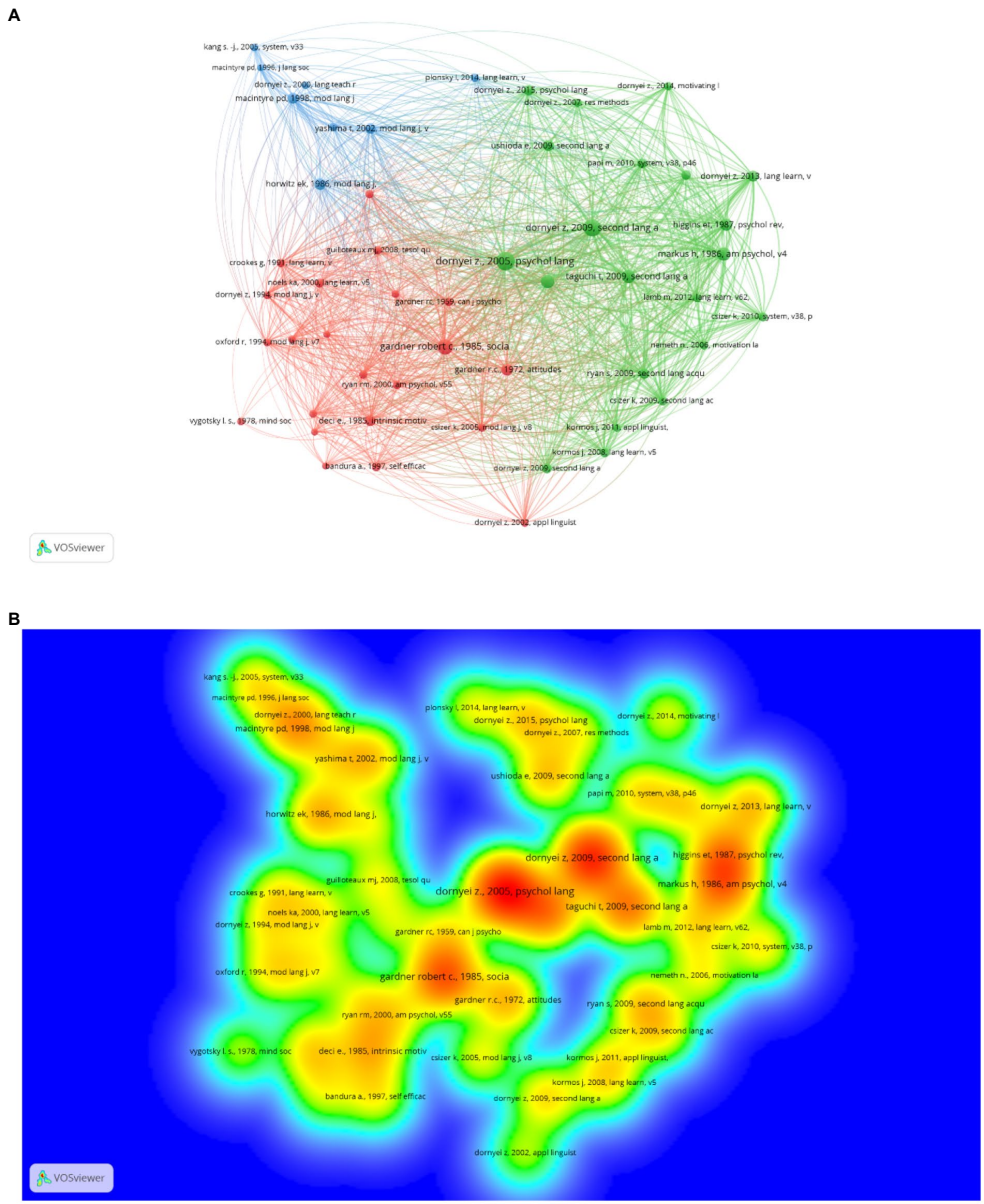
**(A)**. Network map of the most-cited references (2011–2021); **(B)**. Density view (2011–2021).

Among the 4,000+ unique references cited in the articles published between 2000 and 2010, all top 50 most-cited references had citation counts of at least 9. The network map generated by the VOSviewer produced three major clusters (Figure 1A). The density view of the network in Figure 1B is presented to help readers visualize the hot topics between 2000 and 2010. Among the 21,000+ references cited in the articles published between 2011 and 2021, all the top 50 most-cited references were cited at least 26 times. The network analysis produced three major clusters (Figure 2A). Figure 2A shows the connections and clustering of the top references and Figure 2B provides the density view of the network.

It is worth noticing that in the 2000–2010 network map, the three clusters are almost equally distributed, while in the 2011–2021 network map, the size of the L2WTC cluster (the blue cluster in Figure 2A) has shrunk. As a widely accepted model in L2 language learning motivation research, L2WTC is a comprehensive conceptual model proposed by Macintyre and colleagues (Macintyre et al., 1998) with an aim to schematize various traits and situational variables that converge to describe, explain and predict language learners’ willingness to communicate in L2. A large body of research in the 2000–2010 period has been conducted to investigate the underlying factors from this model in different cultural contexts. For instance, Yashima (2002) used this model to examine relations among L2 learning and L2 communication variables in the Japanese English as a foreign language context. In the 2011–2021 period, research began to explore factors that affect or connect to L2WTC. For example, Fallah (2014) developed the L2WTC model based on the research by MacIntyre et al. (1998) and previous empirical studies by examining the potential connections among L2WTC, three individual differences (shyness, motivation, and communication self-confidence) and one situational variable (teacher immediacy). The findings revealed the positive influence of motivation and communication self-confidence on L2WTC, and the indirect influence of shyness and teacher immediacy on L2WTC.

#### The highly influential authors

**Figure 3:**
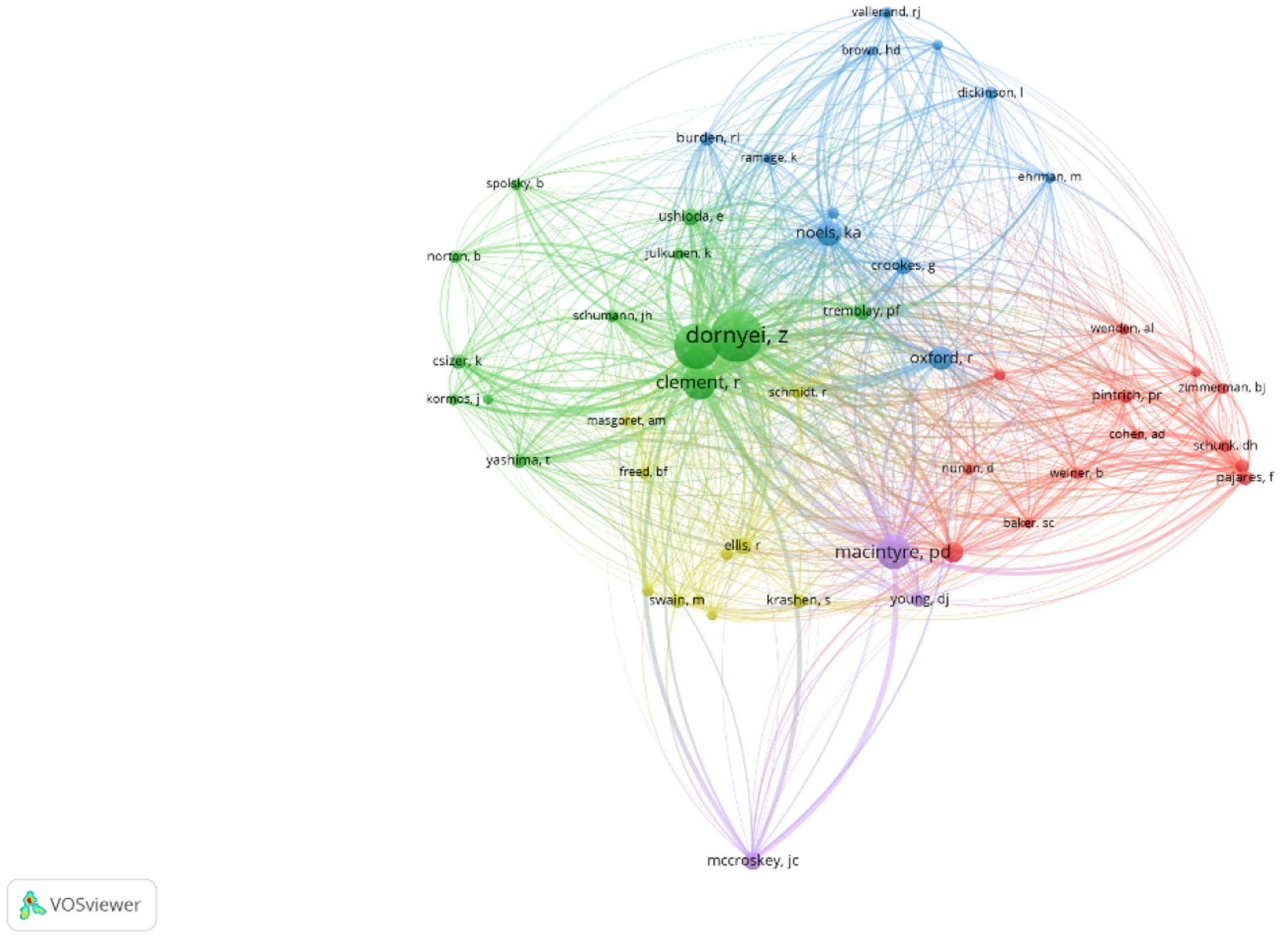
Author network map (2000–2010).

**Figure 4:**
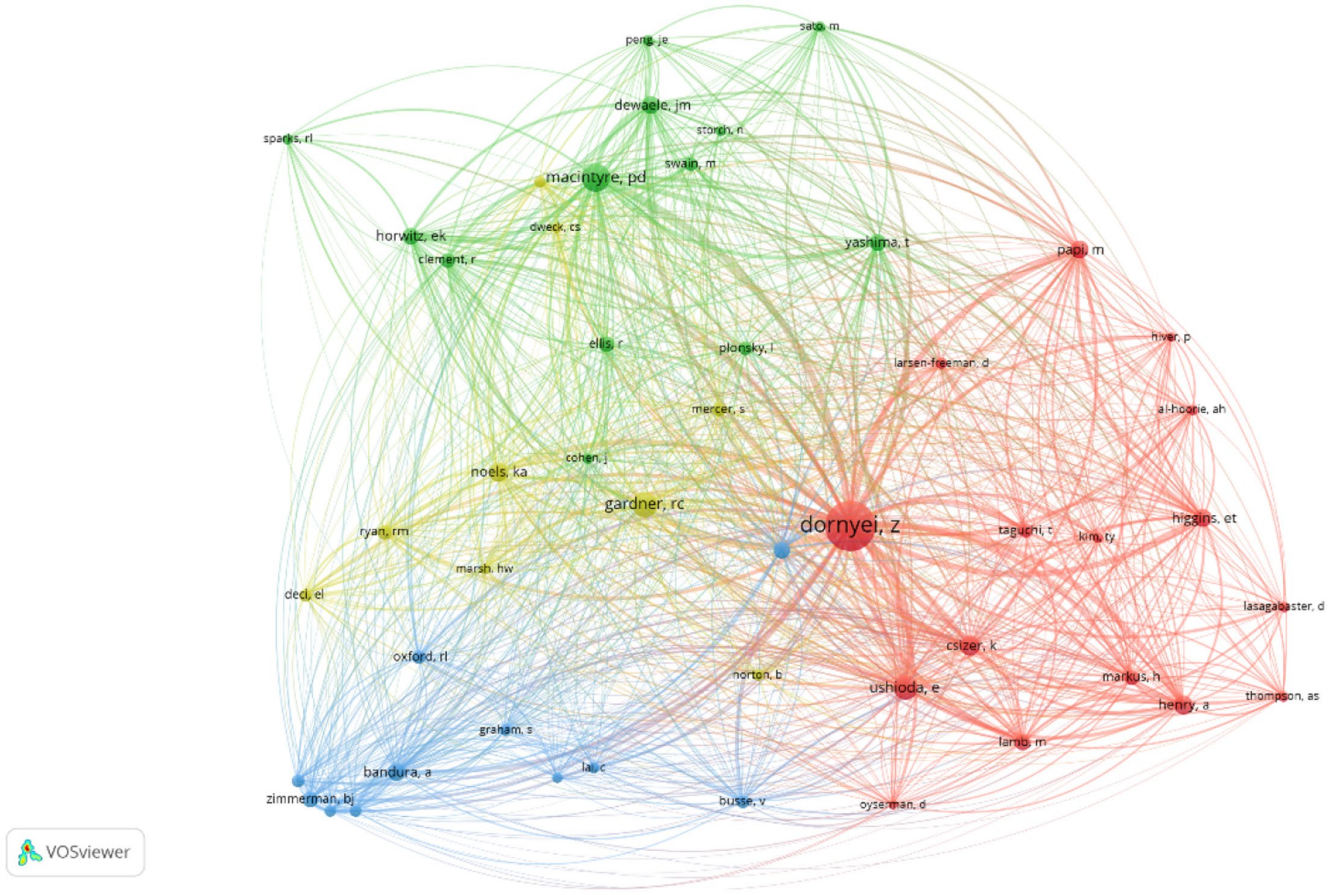
Author network map (2011–2021).

Of the 3,172 authors cited in 2000–2010, more than 10 citations are received by each of the top 50 authors, and of the 12,667 authors cited in 2011–2021, more than 50 citations are received by each of the top 50 authors. The visualizations of the connections among the highly influential authors in the two periods are presented in the networks in Figures 3, 4. As can be seen in Table 2, Zoltán Dörnyei, Robert C. Gardner, and Peter MacIntyre are the top three most-cited authors between 2000 and 2021. Theoretical and empirical studies conducted by the three authors form the backbones of the L2 language learning motivation research. For instance, the classic concept of the integrative motive by Gardner (1985), the L2WTC model by Macintyre et al. (1998), and the theory of L2MSS by Dörnyei (2005) all provide good opportunities to explore and enrich the research territory of motivation in L2 language learning. The lasting influence of those authors has been reflected in the network maps of the cited authors in the two periods as well. For instance, Dörnyei is conspicuously presented in the two network maps (Figures 3, 4).

**Table 2:** Citation of the top 50 most-cited authors between 2000 and 2021.

Author/Citation	Author/Citation	Author/Citation	Author/Citation	Author/Citation
Dörnyei, Z./1586	Henry, A./173	Markus, H./110	Schunk, D./83	Dweck, C./63
Gardner, R./684	Yashima, T./166	Ryan, R./108	Larsen-Freeman, D./75	Marsh, H./63
Macintyre, P./503	Higgins, E./165	Swain, M./105	Crookes, G./73	Masgoret, A./62
Ushioda, E./312	Deci, E./163	Pintrich, P./104	Graham, S./73	Tremblay, P./62
Oxford, R./252	Dewaele, J./162	Ryan, S./95	Busse, V./71	Cohen, J./61
Noels, K./236	Papi, M./154	Mercer, S./94	Lou, N./67	Krashen, S./61
Clément, R./224	Ellis, R./149	Taguchi, T./94	Sparks, R./66	Benson, P./58
Csizer, K./211	Kormos, J./147	Norton, B./87	Lai, C./64	Thompson, A./58
Horwitz, E./190	Lamb, M./136	Pajares, F./87	Long, M./64	Kim, T./57
Bandura, A./177	Zimmerman, B./136	Plonsky, L./86	Mccroskey, J./64	Peng, J./57

As is shown in Figures 3, 4, some influential authors are closely associated with each other, and several clusters have been formed. As is shown in Figure 3, three large clusters have been identified in the 2000–2010 period, including the socio-educational model cluster (in green; e.g., Robert Gardner, Zoltán Dörnyei, Clément Richard, and Ushioda Ema), the self-regulation related cluster (e.g., Paul R. Pintrich, Barry J. Zimmerman, and Frank Pajares), and the Self-Determination Theory (SDT) related cluster (e.g., Kimberly A. Noels, Rebecca Oxford, Robert J. Vallerand).

Three major clusters have also been identified in the network map in the 2011–2021 period. The socio-educational model cluster remains one of the key areas with more authors (e.g., Alastair Henry, Yumiko Taguchi, and Csizer kata) engaging in. It also should be noted that some authors in this cluster have used new methods to explore the topics on L2 motivation. For instance, Papi (2010) takes a structural equation modeling approach to test a theoretical model that covers components in the L2MSS, anxiety, and effort. Another major cluster is formed by authors (e.g., Jean-Marc Dewaele, Peter D. Macintyre, and Tomoko Yashima) who are interested in emotion research, especially those who adopt methods from the field of Positive Psychology (PP). This result demonstrates the importance of positive emotion in L2 language learning. The third cluster that worth noticing is the self-related theory/model cluster, which includes authors such as Barry J. Zimmerman and Rebecca Oxford. As can be seen in Figures 3, 4, the self-regulation-related cluster and the Self-Determination Theory cluster in the 2000–2010 network map have merged into one cluster in the 2011–2021 period.

#### Influential sources of publications

**Figure 5:**
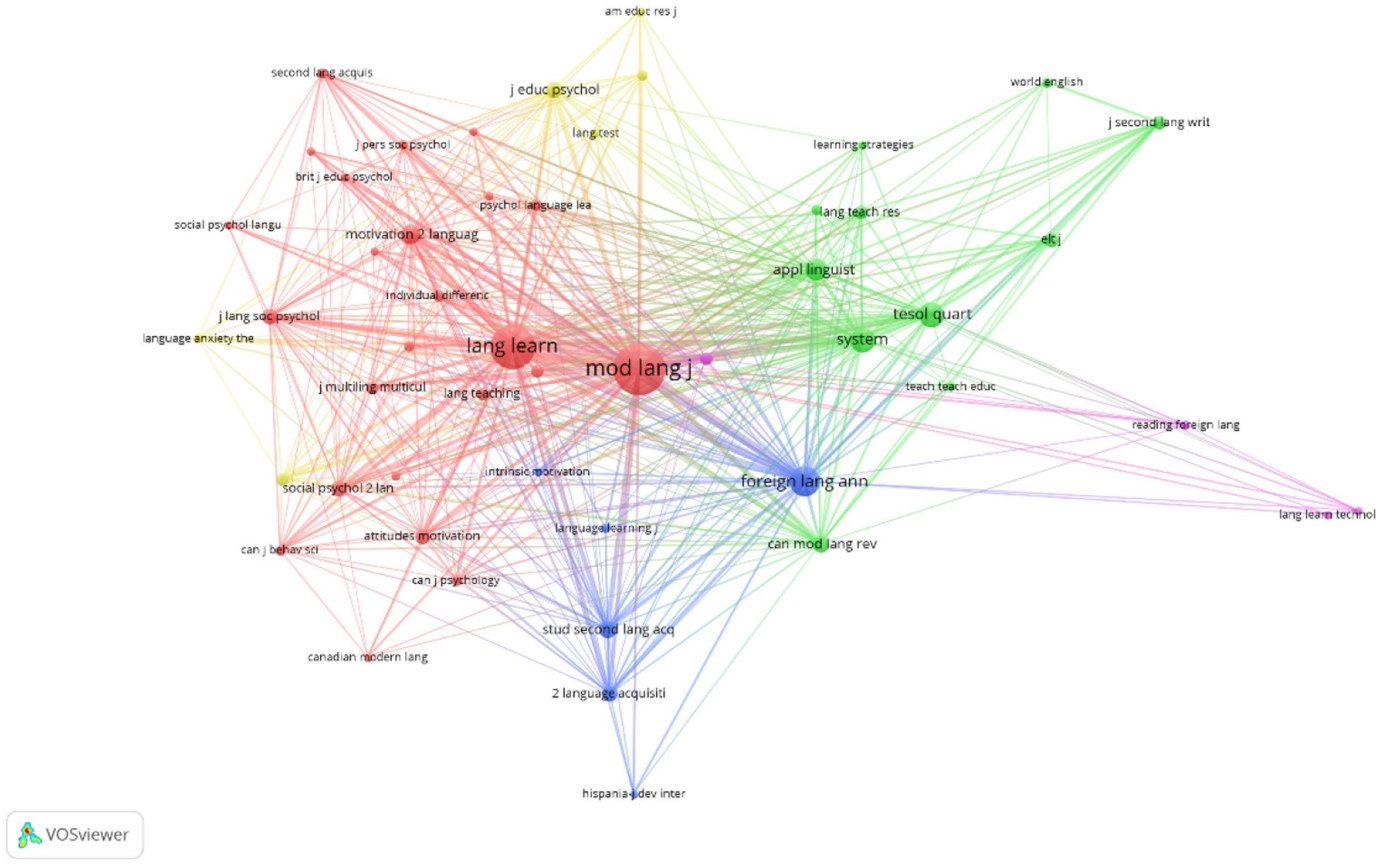
Network map of sources of publications (2000–2010).

**Figure 6:**
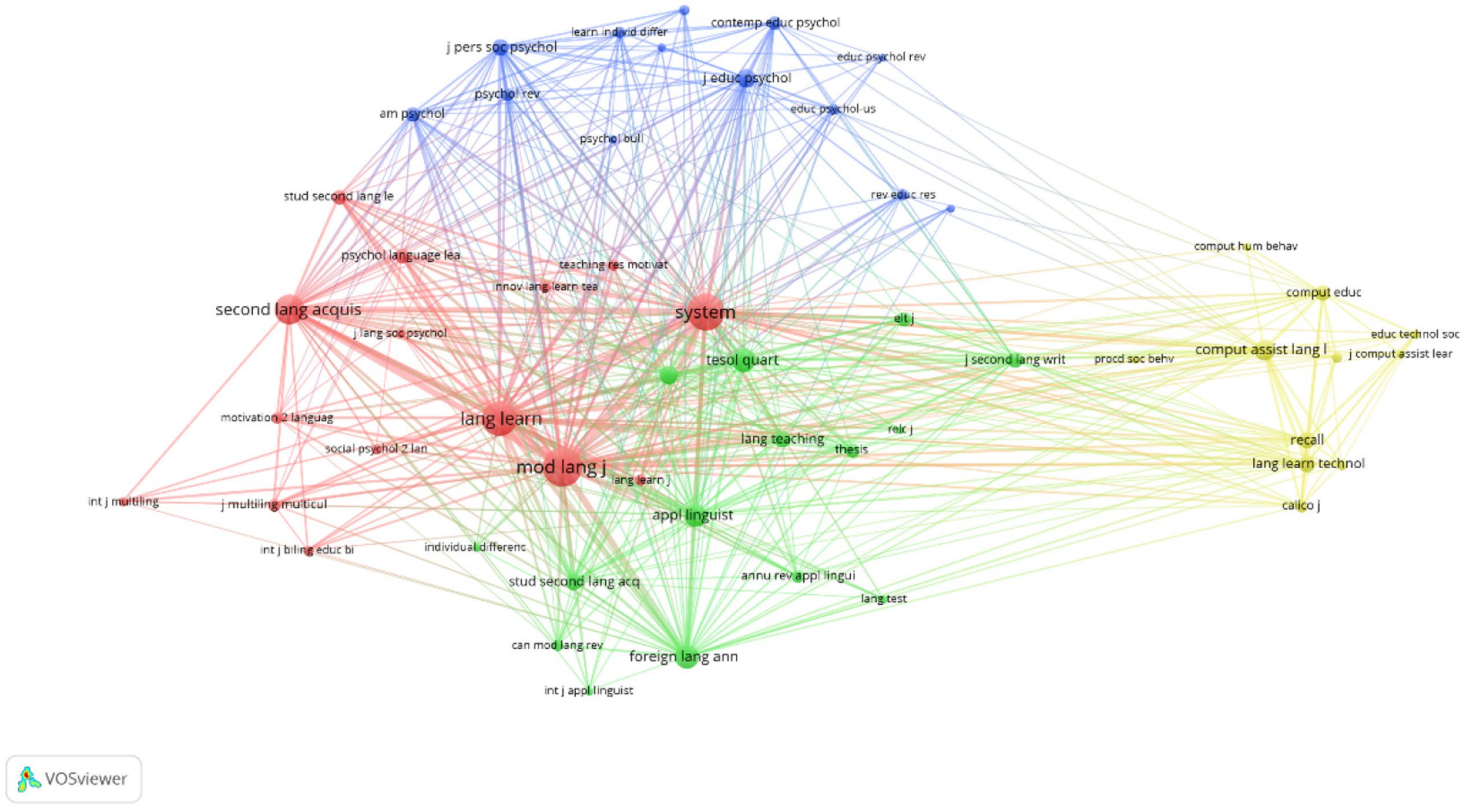
Network map of sources of publications (2011–2021).

Of the 2,429 sources in 2000–2010, around 15 citations are received by each of the top 50 journals/books, and of the 8,042 sources in 2011–2021; more than 70 citations are received by each of the top 50 journals/books. The intellectual structures of the influential sources of publications are visually presented in Figures 5, 6. Three large clusters representing three major sub-areas in the field of the L2 language learning motivation are identified in the period of 2000–2010. The largest cluster (in red) is the second language learning (SLL) cluster, which is represented by the journals of *Modern Language Journal*, *Language Learning*, and two books by Dörnyei (2005), and Gardner and Lambert (1972). The second largest cluster (in green) is the second language teaching (SLT) cluster which includes journals such as *System*, *Applied Linguistics*, *TESOL Quarterly*, *ELT journal*, J*ournal of Second Language Writing*, *World Englishes*, and *Language Teaching Research*. The third cluster (in blue) is the second language acquisition (SLA) cluster, including journals of *Foreign Language Annuals*, *Studies in Second Language Acquisition*, and books such as Second Language Acquisition (Freed, 1995) edited by Freed Barbara.

In the 2022–2021 period, four clusters representing four major sub-areas are identified. The red cluster is labeled as the multilingualism/bilingualism cluster, represented by journals, such as *Journal of Multilingual and Multicultural Development*, *International Journal of Multilingualism*, and *International Journal of Bilingual Education and Bilingualism*. Moreover, journals interested in the concept of multilingualism/bilingualism also exist as an indispensable part of this cluster. For instance, a special issue published by *Modern Language Journal* on the topic of “Beyond Global English: Motivation to Learn Languages in a Multicultural World” (Ushioda and Dörnyei, 2017) has suggested that the dominant status of English as a global language cautions scholars to pay proper regard to notions of self, and identity, which is shaping conceptualizations of second language motivation. The blue cluster is labeled as the educational psychology cluster, including *Journal of Educational Psychology*, *Contemporary Educational Psychology*, and *Journal of Personality and Social Psychology*. The third cluster (in green) is the SLA cluster, including *Applied Linguistics*, *TESOL Quarterly*, *Studies in Second Language Acquisition*, *Annual Review of Applied Linguistics*, and *Foreign Language Annuals*. The last is the technology-based second language learning and teaching (SLLT) cluster (in yellow), including *ReCALL*, *Computer Assisted Language Learning*, *Language Learning and Technology*, and *Journal of Computer Assisted Learning*.

### Keyword analysis

A total token of 15,399 (14,612 extracted from abstracts and 787 supplied by authors) keywords in the 2000–2010 period, and a total token of 40,839 (39,748 extracted from abstracts and 1,091 supplied by authors) keywords in the 2011–2021 period are included for analysis. The results of keyword analysis are presented in this section to provide information about key topics in the L2 motivation research over the past 22 years.

As is shown in Table 3, topics that have gained significantly increasing attention are ideal self, motivational self-system, Chinese EFL learner, and digital game. Topics such as L2 motivational self-system, motivational self, self-image, and possible selves are among the lists of themes with increasing interests over the past 22 years. One plausible reason is that when the emergence of L2MSS model proposed by Dörnyei (2005) shed new insights to the research trajectory of L2 motivation. That is, the social educational approach to L2 motivation by Gardner (1985) has been overshadowed by this new model. Chinese EFL learner is also among the list of topics with significantly increasing interest. One of the reasons may be that an enormous number of EFL learners are Chinese. In addition, Korea, Saudi, and multilingualism are among the list of topics with increasing interest, which indicates that the impact of social environment on second language learning motivation probably varies from one culture to the other. It also should be noted that more and more topics are related to research method (e.g., longitudinal, semi-structured interviews, and multiple regression) over the last 22 years. This increase may be attributed to the surge of publications with a focus on the application of the then emergent theoretical models (e.g., L2WTC and L2MSS). Topics (i.e., digital game and mobile-assisted language learning) related to the use of technology in L2 language learning have attracted more and more researchers’ attention as well. This increase is probably triggered by the effective use of advanced technology (e.g., digital games and computer-assisted pronunciation training) in second language teaching and learning.

**Table 3:** Topics with increasing interests.

Type	Keywords	2000–2010	2011–2021	LL	BIC
**Increase with positive evidence (BIC > 2), strong evidence (BIC > 6.86) or very strong evidence (BIC > 22.22)**					
ad	Ideal self	0	45	28.80	17.86
ad	Motivational self-system	1	41	19.38	8.44
ad	Chinese EFL learner	8	77	16.96	6.03
ad	Digital game	0	21	13.44	2.50
**Increase with evidence (LL > 3.84)**					
au	Longitudinal	1	27	11.24	0.3
ad	Oral proficiency	2	24	15.36	/
au	L2 motivational self-system	1	26	10.67	/
au	Motivation	27	136	10.63	/
ad	Motivational self	2	32	10.45	/
au	Mobile-assisted language learning	0	16	10.24	/
ad	Behavior	2	31	9.93	/
au	L2 motivation	2	29	8.91	/
ad	Engagement	7	50	7.67	/
ad	Korea	4	36	7.39	/
ad	Self-determination	0	11	7.04	/
ad	Self-efficacy belief	0	11	7.04	/
ad	Multilingualism	2	24	6.44	/
ad	Semi-structured interviews	0	10	6.40	/
ad	Development	18	57	5.91	/
au	Autonomy	0	9	5.76	/
ad	Higher education	0	9	5.76	/
ad	Self-regulation	3	26	5.12	/
ad	Self-image	0	7	4.48	/
ad	Writing task	0	7	4.48	/
au	Possible selves	0	7	4.48	/
au	Computer-assisted language learning	1	20	7.35	/
ad	Web	0	7	4.48	/
ad	Beliefs	8	43	3.93	/
ad	Flipped class	0	6	3.84	/
ad	Language instruction	0	6	3.84	/
ad	Language learner motivation	0	6	3.84	/
ad	Multiple regression analysis	0	6	3.84	/
ad	Saudi	0	6	3.84	/
ad	Understanding	6	35	3.80	/

au = author-supplied-keyword; ad = keyword-from-abstracts; LL = Log-likelihood; BIC = Bayes Factor (Szlachta et al., 2012).

BIC values below zero have all been omitted.

The only topic that has experienced a significantly decreasing interest is English as a foreign language. One possible reason for this significant decrease might be that English is not the only choice of foreign languages for learners. Researchers in L2 motivation field have noticed that the theoretical paradigms of L2 language learning motivation should be used to understand the motivation of learning languages other than English (LOTEs; Dörnyei and Al-Hoorie, 2017). For instance, informed by L2MSS, Huang (2019) investigated learners’ motivation for three groups of language (i.e., Southeast Asian, Northeast Asian, and European). Another example is an investigation of the emergence of a multilingual motivational system in a Chinese LOTEs context by Huang and colleagues (Huang et al., 2021).

Though no significant decrease in theoretical paradigms/models in L2 motivation, Table 4 shows that topics on Gardner and integrative motivation are being less touched upon over the past 22 years. The socio-education (SE) model of motivation by Gardner (1985) is one of the most influential models before 1990 and has a lasting effect on current L2 motivation research. However, Gardner’s model has its limitations. L2MSS model of Dörnyei (2003) addresses the limitations of the SE model by adopting the dichotomy of integrative and instrumental orientation, but it “changes the direction of reference from outwardly gauging the target language community to inwardly gauging the future vision of the learner’s self (PP. 577; Huang, 2019).” With exponentially increasing interest in L2MSS model (Al-Hoorie, 2018), Gardner’s SE model has been overshadowed hence.

**Table 4:** Topics with decreasing interests.

Type	Keywords	2000–2010	2011–2021	LL	BIC
**Decrease with positive evidence (BIC > 2), strong evidence (BIC > 6.86) or very strong evidence (BIC > 22.22)**					
ad	English as a foreign language	95	135	20.64	9.70
**Decrease with evidence (LL > 3.84)**					
ad	Integrative motivation	9	3	11.71	0.80
ad	Variable	34	40	11.58	0.64
ad	Attitude	40	57	8.62	/
ad	Canada	5	1	8.19	/
au	Change	20	29	4.1	/
au	Competence	10	9	5.38	/
au	English for special purposes	2	0	5.18	/
ad	Gardner	6	4	4.64	/
ad	Grammar	8	6	5.44	/
ad	instruction	22	46	26.84	/
ad	Interaction	9	48	4.31	/
au	Learner strategies	2	0	5.18	/
ad	Questionnaire	58	104	5.47	/
au	Simulated oral proficiency interview	2	0	5.18	/
au	Task-based instruction	2	0	5.18	/

As for research methods and tools, questionnaire and simulated oral proficiency interview (SOPI) are less frequently used by researchers in L2 motivation research over the past 22 years. One possible reason for this might be that the reliability of the two methods/tools is questioned. For instance, the Greek language the Students’ Motivation Towards Science Learning (SMTSL) questionnaire needs to be adapted if used in a different cultural context (Dermitzaki et al., 2013). As a testing tool for foreign language oral proficiency, the reliability of SOPI ratings at the Intermediate-High through Superior levels is also under question (Stansfield and Kenyon, 1992).

Most research topics of L2 language learning motivation remained stable. Table 5 lists some of the topics without a significant change. Mixed methods remain a popular method probably because it has combined the advantage of both quantitative and qualitative methods to provide multiple perspectives on issues in L2 motivation research. Moreover, topics (e.g., language anxiety, emotion, mindset, and experience) related to propensity factors are also among the list for the reason that those variables are situated and dynamic.

**Table 5:** Topics without much change (2.60 < LL < 3.84).

Type	Keywords	2000–2010	2011–2021	LL	BIC
au	Effectiveness	16	22	3.8	/
ad	Positive attitudes	20	84	3.74	/
ad	Narratives	2	18	3.7	/
ad	Mixed methods	2	18	3.7	/
ad	Secondary school	5	30	3.44	/
ad	Learning environment	4	26	3.44	/
ad	Experience	22	88	3.22	/
au	Second language acquisition	1	12	3.22	/
au	Language anxiety	7	7	3.21	/
au	Agency	0	5	3.2	/
au	Content and language integrated learning	0	5	3.2	/
au	English language learning	0	5	3.2	/
ad	Expectancy	0	5	3.2	/
au	Feedback	0	5	3.2	/
ad	Gender differences	0	5	3.2	/
au	Language mindsets	0	5	3.2	/
au	Pronunciation	0	5	3.2	/
ad	Writing motivation	0	5	3.2	/
ad	Learner motivation	6	33	3.17	/
au	Emotion	3	20	2.76	/
au	Pupil	6	6	2.75	/
au	Activity	17	27	2.61	/
au	Skill	14	21	2.60	/

There is a long list of keywords that remain unchanged, and table only include those topics whose value of LL falls between 2.60 and 3.84.

### Recent trends and future directions

Results from citation analysis, co-citation analysis, and keyword analysis all indicate that motivation is an increasingly important area in second language acquisition. Changes detected between the periods of 2000–2010 and 2011–2021 reflect recent trends in L2 motivation, and those trends may shed light into the directions of a proliferation of future studies in this field. Three major trends are identified from the changes.

First, application and development of existing models/theories will probably become an important area of future research. One typical example is the application and development of the SDT. Results from citation analysis have demonstrated that in the past two decades, SDT has become one of the most established motivational theories in L2 learning and has been robustly applied in multiple cultural settings. For instance, Chen et al. (2021) have applied the basic psychological needs theory and the relationship motivation theory in the Chinese context, Alamer and Lee (2021) in the Saudi Aribian context, Carreira (2012) in the Japanese context, and more commonly in the American (Davis, 2022) and British contexts (Parrish, 2020). Moreover, several mini-theories (e.g., organismic integration theory, cognitive evaluation theory, basic psychological needs theory, and goal contents theory) of SDT have also been generated (Al-Hoorie et al., 2022). In addition, results from co-citation analysis of references reveal the possible trajectory of the development of the existing theoretical models. That is, theoretical models that share common features or involve similar variables may first merge with each other, and a comprehensive synthesis of those features and variables then might provide important insights for the introduction of a new theoretical model in this area.

Second, large datasets will probably be used in a number of future studies, especially review studies. Extracting kernels from wisdom in existing literature, review studies serve as enlightening and guiding beacons for experts and novices interested in the field of L2 motivation research alike. The use of a large dataset in a review study can offer insights to the explanation of the common problems or the description of the shared features in L2 language learning motivation research. Moreover, it also sheds light on the interpretation of results from statistical analysis in a particular discipline. For instance, Yousefi and Mahmoodi (2022) interpreted the overall effectiveness of L2 motivation on language learning by adopting the field-specific interpretation of effect sizes proposed by Plonsky and Oswald (2014) rather than the default one by Cohen (1977).

Third, there is abundant room for further progress in investigating the influence of propensity factors on learners’ motivation. Results from the present study are consistent with Boo et al. (2015) who describe the environment of the L2 motivation research as “dynamic and accommodating, as opposed to one that is static and stagnant” (p. 155). For instance, the rapid advances in technology during 2011 and 2021 play a vital role in the field of L2 motivation research as Golonka et al. (2014) have demonstrated that the application of technological innovations in foreign language learning can increase learners’ interest and motivation and provide them with more interaction opportunities and input of the target language. That is, those propensity factors may affect learners’ motivation if the social contexts or education environments change. Therefore, those topics will probably being investigated in the future.

## Conclusion and implications

This bibliometric study on L2 motivation research has performed analyses on publications, sources of publications, authors, and keywords over the past 22 years. Based on citation information, citation analysis, co-citation analysis, and keyword analysis are performed. Based on the findings in this study, several implications emerged.

First, influential publications, source of references, and authors are identified which may help researchers, especially the newcomers interested in L2 motivation, to effectively screen the massive literature on L2 language learning motivation. The most-cited publications identified from citation analysis can help scholars pinpoint the latest important literature on L2 motivation in the past 22 years, and the highly influential publications identified from co-citation analysis of publications will probably be the classic and key documents in this field. In addition, co-citation analysis of publication sources has presented key journals with similar scope into one cluster, “which may help authors make better decisions concerning which journal(s) may be the best fit for submitting their scholarly research” (Zhang, 2020, p. 218).

Second, the continuity of theoretic development is evident in L2 language learning motivation research. Results from citation analysis, co-citation analysis, and keyword analysis all indicate that theoretical models shift from one period to the other. However, a closer examination of the most-cited publications and the highly influential documents reveals that the fresh theoretical models are developed or expanded on the basis of the existing classic theories. By maintaining the best of the existing theories and pushing the relevant parameters outward, a more comprehensive model might be eventually created (Oxford and Shearin, 1994).

Last, an air of active use of qualitative approaches has been detected in L2 language learning motivation research in the last 22 years. Boo et al. (2015) have suggested that quantitative approaches would probably lose their dominant role in the field of L2 language learning motivation. Results of the keyword analysis in this study indicate that qualitative approaches such as semi-structured interviews are receiving an increasing interest. Moreover, mixed methods remain a stable topic in this field. This finding probably indicates that the field of L2 motivation research is in the phase of the dynamic turn (Boo et al., 2015). More qualitative or combined approaches should be applied in research on L2 motivation.

Future directions in L2 motivation research have also been identified. However, as Boo et al. (2015) have pointed out that motivation research in the field of second language acquisition is in the state of dynamic development, it should be reminded that those directions may offer insights for studies in the near future, and there are still uncertainties when using them to predict longer-term future development in this field.

## Data availability statement

The original contributions presented in the study are included in the article/supplementary material, further inquiries can be directed to the corresponding author.

## Author contributions

The author confirms being the sole contributor of this work and has approved it for publication.

## Funding

The research work reported in this article was supported by the China Foreign Language Education Foundation (Grant number: ZGWYJYJJ11Z035).

## Conflict of interest

The author declares that the research was conducted in the absence of any commercial or financial relationships that could be construed as a potential conflict of interest.

## Publisher’s note

All claims expressed in this article are solely those of the authors and do not necessarily represent those of their affiliated organizations, or those of the publisher, the editors and the reviewers. Any product that may be evaluated in this article, or claim that may be made by its manufacturer, is not guaranteed or endorsed by the publisher.

## Footnotes

^1^ http://ucrel.lancs.ac.uk/llwizard.html
